# Evaluating metabolites in patients with major depressive disorder who received mindfulness-based cognitive therapy and healthy controls using short echo MRSI at 7 Tesla

**DOI:** 10.1007/s10334-016-0526-7

**Published:** 2016-02-09

**Authors:** Yan Li, Angela Jakary, Erin Gillung, Stuart Eisendrath, Sarah J. Nelson, Pratik Mukherjee, Tracy Luks

**Affiliations:** Department of Radiology and Biomedical Imaging, University of California, San Francisco, Radiology Box 2532, Byers Hall, 1700 4th Street, San Francisco, CA 94158-2532 USA; Department of Psychiatry, University of California, San Francisco, San Francisco, CA USA; Department of Bioengineering and Therapeutic Sciences, University of California, San Francisco, San Francisco, CA USA

**Keywords:** Major depressive disorder, MBCT, Magnetic resonance spectroscopic imaging, 7 Tesla

## Abstract

**Objectives:**

Our aim was to evaluate differences in metabolite levels between unmedicated patients with major depressive disorder (MDD) and healthy controls, to assess changes in metabolites in patients after they completed an 8-week course of mindfulness-based cognitive therapy (MBCT), and to exam the correlation between metabolites and depression severity.

**Materials and methods:**

Sixteen patients with MDD and ten age- and gender-matched healthy controls were studied using 3D short echo-time (20 ms) magnetic resonance spectroscopic imaging (MRSI) at 7 Tesla. Relative metabolite ratios were estimated in five regions of interest corresponding to insula, anterior cingulate cortex (ACC), caudate, putamen, and thalamus.

**Results:**

In all cases, MBCT reduced severity of depression. The ratio of total choline-containing compounds/total creatine (tCr) in the right caudate was significantly increased compared to that in healthy controls, while ratios of *N*-acetyl aspartate (NAA)/tCr in the left ACC, myo-inositol/tCr in the right insula, and glutathione/tCr in the left putamen were significantly decreased. At baseline, the severity of depression was negatively correlated with my-inositol/tCr in the left insula and putamen. The improvement in depression severity was significantly associated with changes in NAA/tCr in the left ACC.

**Conclusions:**

This study has successfully evaluated regional differences in metabolites for patients with MDD who received MBCT treatment and in controls using 7 Tesla MRSI.

## Introduction

Major depressive disorder (MDD) affects about 6.7 % of the adults in the United States per year and is the leading cause of disability for people from 15 to 44 years old. The most common treatments include antidepressant medications and psychotherapy. Most patients with MDD have recurrent episodes and often experience treatment failure under current therapies, which severely impact their quality of life. Mindfulness-based cognitive therapy (MBCT), a method for integrating cognitive behavioral therapy with mindfulness meditation, teaches patients how to disengage from habitual “automatic” dysfunctional cognitive routines, in particular depression-related ruminative thought patterns. MBCT was first used for preventing the relapse of depression [1] and has shown its efficacy in reducing depressive recurrence [2] and treating acute [3], chronic [4], and treatment-resistant depression [5] as an augmentation. These findings make MBCT treatment a potential valuable stand-alone treatment for MDD.

Although the etiology and neuropathology of MDD is still not fully understood, it is thought to be associated with chemical changes that involve multiple brain circuits. Proton magnetic resonance spectroscopy (MRS) is a powerful tool for noninvasively investigating brain metabolism. Spectra acquired with long echo times (TEs) provide estimates of metabolite levels, such as total choline-containing compounds (tCho, reflecting membrane synthesis), total creatine (tCr, reflecting cellular bioenergetics), and *N*-acetyl aspartate (NAA, a neuronal marker). At short TEs, metabolites such as myo-inositol (mI), glutamate (Glu), and glutamine (Gln) also appear, but overlap between peaks can make it difficult to obtain accurate quantification. Spectral editing methods using J-coupling differences allow unobstructed detection of glutathione (GSH) and γ-aminobutyric acid (GABA) but require acquisition from a relatively large region and/or relatively long acquisition time. High field MR systems, such as 7 Tesla (7T) scanners, offer advantages in higher signal-to-noise ratio (SNR) and enhanced spectral quantification for all of these metabolites [6]. Other brain metabolites, such as *N*-acetyl-aspartyl glutamate (NAAG), glycine (Gly), and glucose that have much lower concentration and/or overlap with the major peaks, can also be detected at high field using ultrashort TE (≤10 ms) MRS or TE-optimized sequences [7–9].

Multiple studies using single-voxel and spectroscopic imaging have been performed at lower field strengths in patients with MDD and have reported regional and/or global metabolite differences. Although the overall consensus is that there is hypometabolism in patients compared to healthy controls, there was some variability in the results obtained [10]. Efficacy of other MDD treatments was evaluated using MRS in different brain regions. Changes in metabolites, such as Glu, NAA, and tCho, were found to be correlated with response to pharmacotherapy or antidepressant stimulation techniques [11]. Regions of interest (ROI) for these studies involved locations in the basal ganglia, limbic system, frontal cortex, and occipital cortex. Multivoxel 3D magnetic resonance spectroscopic imaging (MRSI) is of interest because it allows characterization of the spatial extent and metabolic properties within multiple regions of the brain.

The purpose of this study was to compare relative levels of metabolites between unmedicated patients with MDD and healthy controls, to evaluate the relationship between metabolite levels and disease severity, and to examine the association between changes in metabolite levels and the outcome of completed MBCT treatment for these patients using 3D short echo-time MRSI at 7T. The patient population comprised a subset of individuals who participated in a previously published project designed to evaluate the efficacy of treatment based upon standard clinical criteria [12]. ROIs within the basal ganglia, limbic system, and frontal cortex were selected for analysis because they have been identified as being relevant for studying psychiatric diseases previously.

## Materials and methods

### Study population

Sixteen depressed patients (11 F/5 M, 30 ± 7 years) who met the* Diagnostic and Statistical Manual of Mental Disorders, Fourth Edition *(DSM-IV) criteria for MDD and who had been medication free for at least 6 weeks, and ten age- and gender-matched healthy controls (7 F/3 M, 32 ± 9 years) who had no history of neurologic illness, traumatic brain injury, or DSM-IV Axis I or II diagnosis were recruited into this study. Individuals with alcohol or substance abuse or dependence within the last 3 months, inadequate English language comprehension, or significant medical conditions or contraindications to MR systems were excluded, as were individuals who currently meditated once or more a week or who practiced yoga twice or more per week.

Each participant was given written informed consent in accordance with the University of California San Francisco (UCSF) IRB procedures. Baseline and post-MBCT MRSI data were successfully acquired from nine patients. Five additional patients had baseline MRSI data only. Among these five patients, two dropped out of the study during MBCT treatment, one declined to participate in the post-MBCT MR scans, and two became pregnant during MBCT treatment. Two patients only had post-MBCT MRSI scans for purely logistical reasons.

Depressive symptoms were evaluated using the Hamilton Depression Severity Rating 17-item scale (HAMD-17) in all participants at baseline. Fourteen of the 16 patients completed the manual and were reassessed using the HAMD-17 within 2 weeks of completing treatment (post-MBCT). Remission was identified by scores ≤7 on the post-MBCT HAMD-17, and the reduction rate was calculated by changes between baseline and post-MBCT HAMD-17 scores divided by the baseline scores.

### Mindfulness-based cognitive therapy (MBCT) methods

MBCT treatment consisted of 8 weekly classes each lasting 2.25 h, and participants were also asked to complete 45 min of homework 6 days per week. This 8-week MBCT group intervention used a clinician and participant manual developed by Segal et al. [13] with modifications for current depression, and was described in Eisendrath et al. [14].

### MR acquisitions

All MR scans were performed using a 32-channel receive-only array with a volume-transmit head coil (NOVA Medical, Wilmington, MA, USA) on a GE 7T MR950 scanner (GE Healthcare, Waukesha, WI, USA). Anatomical imaging consisted of a T1-weighted sagittal scout (TR/TE = 6/2 ms), 3D T1-weighted inversion recovery-prepared spoiled gradient echo (IR SPGR) [TR/TE/inversion time (TI) = 6/2/600 ms, matrix size = 256 × 256 × 192 FOV = 256 × 256 × 192 mm^3^, voxel size = 1 × 1 × 1 mm^3^), and 3D T2-weighted fast spin echo (FSE-Cube) (TR/TE = 6000/60 ms, matrix size = 512 × 512, FOV = 256 × 256 mm^2^, voxel size = 0.5 × 0.5 mm^2^, slice thickness/overlap = 2/1 mm, acceleration factor = 3) images.

**Fig. 1:**
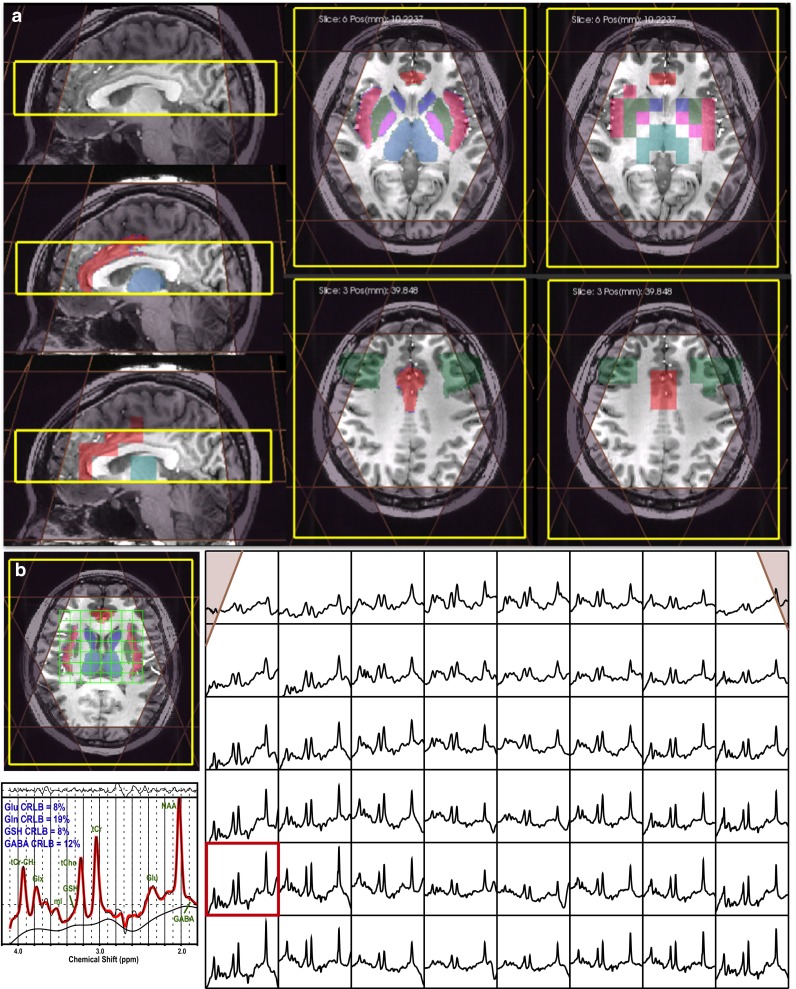
**a** Location of 3D magnetic resonance spectroscopic imaging (MRSI) prescription and regions of interest (ROIs) that were segmented using Harvard–Oxford cortical and subcortical atlases, inversely transformed from the MNI space and then resampled to the resolution of spectra overlaid on T1-weighted images. **b** MRSI data (with baseline between 1.6 and 4.2 ppm) from a patient with major depressive disorder (MDD) and an example of fitted spectra from LCModel in the selected voxel

Optimization of high order shimming and transmit gains were performed prior to spectral data acquisitions. 3D H-1 MRSI was obtained using spectrally selective adiabatic inversion recovery lipid suppression, chemical shift selective (CHESS) water suppression, automatically prescribed very selective suppression (VSS) outer volume suppression, and spin-echo slice selection with TE/TR = 20/2000 ms, spectra array = 16–20 × 22 × 8, and nominal spatial resolution = 1 cm^3^ [15]. Total acquisition time was 8.77 min for 16 × 22 × 8 or 10.96 min for 20 × 22 × 8 spectral arrays when applying an interleaved flyback echo-planar trajectory [16] in the anterior/posterior direction of the FOV. Slice direction of spectra data was placed parallel to the anterior commissure (AC)–posterior commissure (PC) line with full coverage of the thalamus (Fig. 1a). The variation in radio-frequency (RF) excitation due to B1 inhomogeneities was minimized by adjusting both the transmitter gain and relative RF power of CHESS, 90°, and 180° pulses.

### MR postprocessing

Postprocessing was performed using a previously published methodology [15]. Spectral arrays acquired with the interleaved flyback readout trajectory were combined and reordered to a rectilinear grid [16], processed with phase and frequency corrections individually for each coil, combined by weighting with coil sensitivities, and then quantified using LCModel [17]. Metabolite signals for the basis set were generated using NMR-SCOPE [18] with prior knowledge of chemical shift and J-coupling information [19]. Only those voxels with relative Cramer–Rao lower bounds (CRLBs) <10 % for tCho, tCr, and NAA, and 20 % for Glu, Gln, GABA, mI, Gly, and GSH, were included in the analysis.

The 3D MRSI data were referenced to the 3D T1-weighted images by assuming that there was no movement between the two acquisitions. Segmentation of the brain was performed automatically on these images using Harvard–Oxford cortical and subcortical structural atlases [20, 21]. ROIs used in the analysis of the 3D MRSI consisted of insula, medial frontal gyrus (MFG), anterior cingulate cortex (ACC), caudate, putamen, pallidum, and thalamus. The segmented ROIs were then aligned to the orientation of the 3D MRSI and down-sampled to the resolution of spectral data. Median metabolite ratios from voxels overlapping by at least 40 % with the anatomical ROIs were included in the analysis. An example of ROIs overlaid on the 3D T1-weighted images is shown in Fig. 1a. The signal from the MFG was partially suppressed by the outer-volume suppression bands; the small size of the pallidum meant that overlapping voxels may contain a relatively high portion of other tissue. These two regions were therefore excluded from further analysis. The remaining five ROIs were analyzed and divided into right- and left-hemisphere sides.

### Statistics

Statistical analysis was performed using R (www.r-project.org). Given the small sample size of the study population, nonparametric statistic methods were used for data analysis, and the number of participants in each group was required to be more than seven. Adjustment for multiple comparisons was not performed due to the exploratory nature of the study. A *p* value of <0.05 was considered as significant at this stage. Wilcoxon signed-rank tests were used to test differences in HAMD-17 scores between baseline and post-MBCT evaluations. Differences in baseline metabolite ratios between patients and controls were assessed using Wilcoxon rank-sum tests, and the effects of MBCT on metabolite ratios were assessed using Wilcoxon signed-rank tests. Spearman rank-correlation coefficients were calculated to determine the association between baseline metabolite ratios and baseline HAMD-17 scores, between baseline metabolite ratios and reduction rates on HAMD-17 scores, and between changes in metabolite ratios and reduction rates in HAMD-17 scores.

## Results

### Participant characteristics and treatment outcomes

**Fig. 2:**
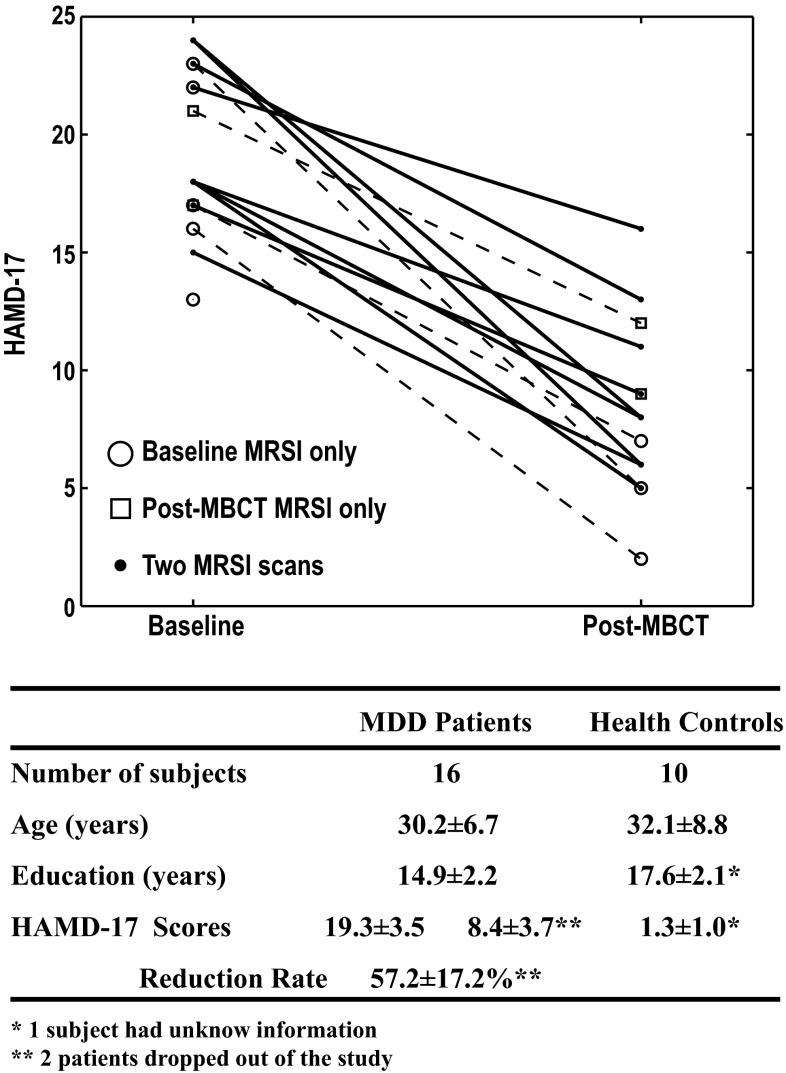
Characteristics of study participants. Age, education, and Hamilton Depression Severity Rating 17-item scale (HAMD-17) scores are summarized for patients and healthy controls. All patients demonstrated reduced HAMD-17 scores after completing mindfulness-based cognitive therapy (MBCT). HAMD-17 scores for those who did or did not have 3D magnetic resonance spectroscopic imaging (MRSI) scans are shown in different shapes of markers

The characteristics of study participants are summarized in Fig. 2, which also shows changes in HAMD-17 in all patients. At baseline, the median HAMD-17 for patients was 18 (range 13–24) and declined to 8 (range 2–18) after the 8-week MBCT treatment. All 14 patients who completed the MBCT treatment demonstrated reduced HAMD-17 scores, six achieved remission, and eight had a reduction on the HAMD-17 score >50 %. The difference in HAMD-17 scores between baseline and post-MBCT was highly significant (*p* < 0.001).

### MR characteristics

The presence of obvious imaging abnormalities was ruled out based on FSE-Cube images by a neuroradiologist. An example of 3D MRSI data from a patient and the ROIs segmented using the Harvard–Oxford cortical and subcortical structural atlases are illustrated in Fig. 1b. Note that the baseline was not removed from the spectra shown and that the CRLB estimates of reliability of Glu, GSH, and mI measures obtained from LCModel were relatively small.

### Differences in metabolite ratios between groups

**Fig. 3:**
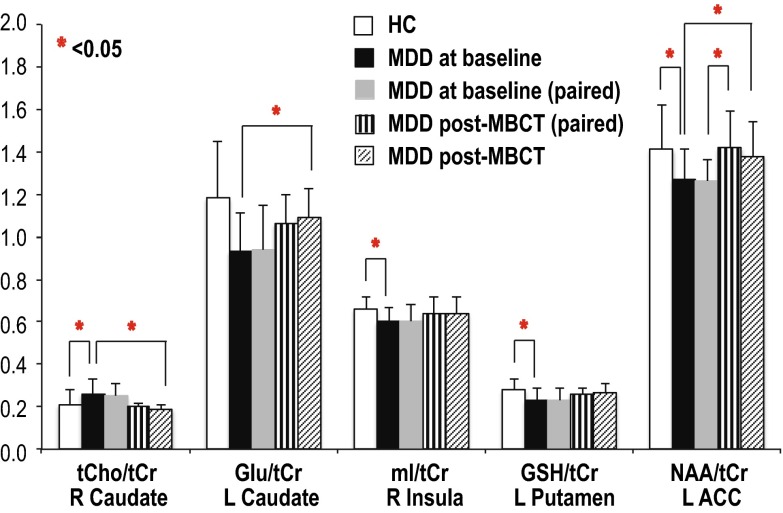
Significant differences in metabolite ratios between patients with major depressive disorders (MDD) and controls at baseline and between patients at baseline and after mindfulness-based cognitive therapy (MBCT) in the R caudate, L caudate, R insula, L putamen, and L anterior cingulate cortex (ACC). (*R* right; *L* left)

Figure 3 summarizes ROIs that had significant differences in metabolite ratios between groups. No differences were detected in the thalamus.

Table 1 gives median and interquartile range (IQR) for tCho/tCr, NAA/tCr, mI/tCr, Glu/tCr, GABA/tCr, and GSH/tCr in each ROI from patients and controls at baseline. When compared to values in controls, metabolite ratio levels of the following were significantly lower at baseline in patients: mI/tCr in the right insula [patients vs. controls, mean ± standard deviation (SD), 0.60 ± 0.07 vs. 0.66 ± 0.06, *p* = 0.042], GSH/tCr in the left putamen (0.23 ± 0.06 vs. 0.28 ± 0.05, *p* = 0.044), NAA/tCr in the left ACC (1.27 ± 0.14 vs. 1.41 ± 0.21, *p* = 0.044). The level of tCho/tCr in the right caudate (0.26 ± 0.07 vs. 0.21 ± 0.07, *p* = 0.041) was significantly higher at baseline in patients with MDD than in controls.

**Table 1:** Metabolite ratios [median (interquartile range (IQR)] within regions of interest (ROIs) in patients with MDD and healthy controls (HC) at baseline

tCho/tCr		NAA/tCr		mI/tCr		Glu/tCr		GABA/tCr		GSH/tCr	
MDD	HC	MDD	HC	MDD	HC	MDD	HC	MDD	HC	MDD	HC
Left insula											
0.20 (0.03)	0.21 (0.03)	1.19 (0.11)	1.32 (0.24)	0.65 (0.07)	0.64 (0.07)	1.14 (0.12)	1.21 (0.21)	0.28 (0.08)	0.28 (0.04)	0.28 (0.08)	0.34 (0.04)
Right insula											
0.22 (0.02)	0.22 (0.03)	1.25 (0.21)	1.25 (0.20)	**0.61 (0.11)**	**0.68 (0.09)**	1.21 (0.15)	1.29 (0.15)	0.30 (0.14)	0.30 (0.08)	0.29 (0.04)	0.34 (0.06)
Left ACC											
0.23 (0.04)	0.24 (0.04)	**1.22 (0.18)**	**1.32 (0.25)**	0.68 (0.14)	0.69 (0.23)	1.31 (0.20)	1.44 (0.17)	0.32 (0.10)	0.40 (0.16)	0.27 (0.08)	0.23 (0.04)
Right ACC											
0.24 (0.05)	0.24 (0.03)	1.22 (0.25)	1.29 (0.30)	0.68 (0.12)	0.72 (0.15)	1.29 (0.11)	1.36 (0.16)	0.37 (0.10)	0.40 (0.16)	0.24 (0.03)	0.27 (0.07)
Left caudate											
0.23 (0.09)	0.21 (0.04)	1.18 (0.20)	1.22 (0.14)	0.56 (0.19)	0.50 (0.24)	0.96 (0.25)	1.16 (0.38)	0.34 (0.12)	0.31 (0.12)	0.30 (0.10)	0.29 (0.05)
Right caudate											
** 0.25 (0.05)**	**0.21 (0.05)**	1.18 (0.40)	1.17 (0.46)	0.57 (0.15)	0.49 (0.16)	1.05 (0.40)	0.87 (0.25)	0.37 (0.11)	0.32 (0.12)	0.27 (0.12)	0.30 (0.12)
Left putamen											
0.19 (0.06)	0.22 (0.03)	1.22 (0.29)	1.34 (0.39)	0.48 (0.12)	0.50 (0.11)	1.20 (0.23)	1.02 (0.14)	0.30 (0.17)	0.32 (0.08)	**0.22 (0.09)**	**0.30 (0.05)**
Right putamen											
0.22 (0.07)	0.25 (0.04)	1.38 (0.35)	1.35 (0.36)	0.44 (0.13)	0.50 (0.18)	1.08 (0.30)	0.90 (0.18)	0.29 (0.11)	0.27 (0.21)	0.28 (0.07)	0.32 (0.08)
Left thalamus											
0.23 (0.03)	0.24 (0.04)	1.42 (0.11)	1.46 (0.23)	0.61 (0.08)	0.68 (0.22)	1.04 (0.12)	1.02 (0.07)	0.32 (0.05)	0.34 (0.10)	0.22 (0.03)	0.21 (0.03)
Right thalamus											
0.24 (0.04)	0.22 (0.04)	1.36 (0.24)	1.45 (0.45)	0.62 (0.15)	0.71 (0.11)	1.15 (0.21)	1.22 (0.33)	0.33 (0.04)	0.36 (0.01)	0.23 (0.04)	0.24 (0.04)

Metabolite values in bold were significant between MDD and HC

*MDD* major depressive disorder, *tCho/tCr* total choline-containing compounds/total creatine, *NAA N*-acetyl aspartate, *Gly* glycine,* mI* myo-inositol, *Glu* glutamate, *Gln* glutamine, *GSH* glutathione, *GABA* γ-aminobutyric acid

Table 2 demonstrates median and IQR of tCho/tCr, NAA/tCr, mI/tCr, Glu/tCr, GABA/tCr, and GSH/tCr from patients at baseline and post-treatment. After completing MBCT treatment, the levels of NAA/tCr in the left ACC were significantly increased (1.26 ± 0.10 vs. 1.42 ± 0.17, signed-rank, *p* = 0.018). Although tCho/tCr in the right caudate (0.25 ± 0.06 vs. 0.20 ± 0.02, signed-rank, *p* = 0.084) were not significantly different from that at baseline in patients who had both baseline and post-MBCT MRSI scans (nine patients), they were statistically decreased (0.26 ± 0.07 vs. 0.19 ± 0.02, rank-sum, *p* = 0.004) when comparing all patients between baseline and post-MBCT (14 vs. 11 patients). Glu/tCr in the left caudate (0.93 ± 0.18 vs. 1.09 ± 0.14, rank-sum, *p* = 0.026), mI/tCr in right caudate (0.62 ± 0.14 vs. 0.48 ± 0.07, rank-sum, *p* = 0.017), and tCho/tCr in the right putamen (0.22 ± 0.04 vs. 0.18 ± 0.02, rank-sum, *p* = 0.011) were also significantly different from baseline to post-treatment.

**Table 2:** Median [interquartile range (IQR)] of metabolite ratios within regions of interest (ROIs) in patients with MDD at baseline and post-treatment

tCho/tCr		NAA/tCr		mI/tCr		Glu/tCr		GABA/tCr		GSH/tCr	
Pre	Post	Pre	Post	Pre	Post	Pre	Post	Pre	Post	Pre	Post
Left insula											
0.20 (0.03)	0.19 (0.03)	1.19 (0.11)	1.24 (0.09)	0.65 (0.07)	0.67 (0.08)	1.14 (0.12)	1.25 (0.29)	0.28 (0.08)	0.30 (0.07)	0.28 (0.08)	0.29 (0.05)
Right insula											
0.22 (0.02)	0.20 (0.04)	1.25 (0.21)	1.24 (0.11)	0.61 (0.11)	0.63 (0.12)	1.21 (0.15)	1.32 (0.22)	0.30 (0.14)	0.33 (0.08)	0.29 (0.04)	0.33 (0.07)
Left ACC											
0.23 (0.04)	0.24 (0.05)	**1.22 (0.18)**	**1.31 (0.17)**	0.68 (0.14)	0.70 (0.13)	1.31 (0.20)	1.40 (0.24)	0.32 (0.10)	0.36 (0.10)	0.27 (0.08)	0.25 (0.03)
Right ACC											
0.24 (0.05)	0.26 (0.07)	1.22 (0.25)	1.33 (0.09)	0.68 (0.12)	0.75 (0.12)	1.29 (0.11)	1.45 (0.20)	0.37 (0.10)	0.37 (0.10)	0.24 (0.03)	0.27 (0.05)
Left caudate											
0.23 (0.09)	0.19 (0.05)	1.18 (0.20)	1.24 (0.31)	0.56 (0.19)	0.49 (0.10)	**0.96 (0.25)**	**1.10 (0.18)**	0.34 (0.12)	0.36 (0.19)	0.30 (0.10)	0.29 (0.10)
Right caudate											
** 0.25 (0.05)**	**0.20 (0.03)**	1.18 (0.40)	1.13 (0.24)	**0.57 (0.15)**	**0.46 (0.09)**	1.05 (0.40)	1.09 (0.16)	0.37 (0.11)	0.30 (0.05)	0.27 (0.12)	0.30 (0.13)
Left putamen											
0.19 (0.06)	0.19 (0.04)	1.22 (0.29)	1.22 (0.11)	0.48 (0.12)	0.51 (0.11)	1.20 (0.23)	1.05 (0.05)	0.30 (0.17)	0.34 (0.16)	0.22 (0.09)	0.26 (0.03)
Right putamen											
** 0.22 (0.07)**	**0.18 (0.02)**	1.38 (0.35)	1.14 (0.08)	0.44 (0.13)	0.45 (0.11)	1.08 (0.30)	1.00 (0.24)	0.29 (0.11)	0.27 (0.09)	0.28 (0.07)	0.29 (0.06)
Left thalamus											
0.23 (0.03)	0.23 (0.04)	1.42 (0.11)	1.44 (0.26)	0.61 (0.08)	0.59 (0.15)	1.04 (0.12)	1.05 (0.18)	0.32 (0.05)	0.30 (0.05)	0.22 (0.03)	0.23 (0.07)
Right thalamus											
0.24 (0.04)	0.22 (0.04)	1.36 (0.24)	1.46 (0.27)	0.62 (0.15)	0.60 (0.15)	1.15 (0.21)	1.10 (0.21)	0.33 (0.04)	0.28 (0.09)	0.23 (0.04)	0.24 (0.06)

Metabolite values in bold were significantly different between baseline and post-treatment

*MDD* major depressive disorder, *tCho/tCr* total choline-containing compounds/total creatine, *NAA N*-acetyl aspartate, *Gly* glycine,* mI* myo-inositol,* Glu* glutamate,* Gln* glutamine,* GSH* glutathione,* GABA* γ-aminobutyric acid

### Correlations between metabolite ratios and HAMD-17 scores

**Fig. 4:**
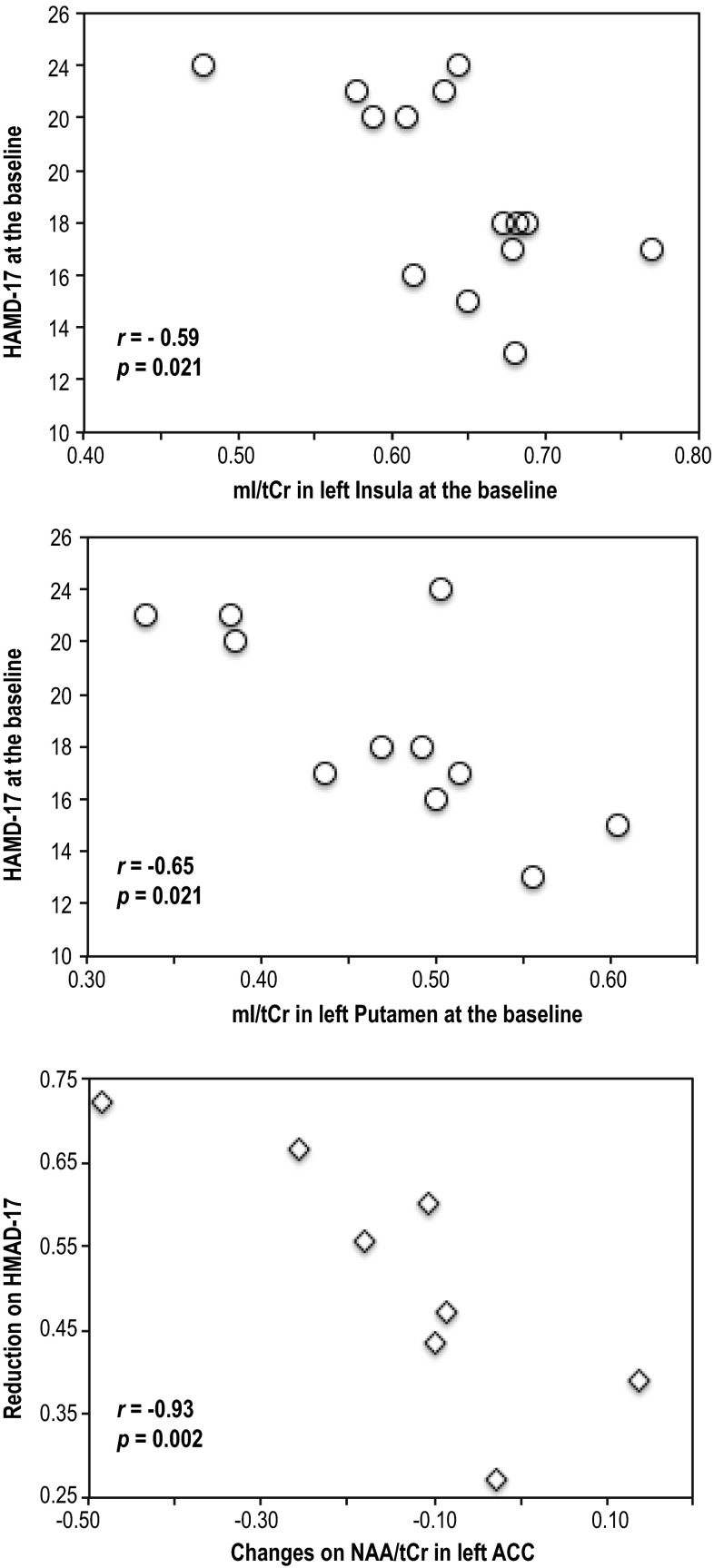
Significant associations between metabolite ratios and Hamilton Depression Severity Rating 17-item scale (HAMD-17) scores at baseline in patients (*top, middle*) and between percent changes on metabolite ratios and reduction on HAMD-17 scores after completing MBCT (*bottom*). The correlation coefficients and *p* values were calculated using Spearman rank tests

Figure 4 shows the regions in which there were significant associations between metabolite ratios and HAMD-17 scores. Higher HAMD-17 scores at baseline were significantly associated with lower levels of mI/tCr in the left insula (correlation coefficient, *r* = −0.59, *p* = 0.026, *N* = 14) and left putamen (*r* = −0.65, *p* = 0.021, *N* = 12) at baseline. Increases in NAA/tCr from baseline to post-MBCT were significantly associated with a reduction in HAMD-17 scores after completing MBCT in the left ACC (*r* = −0.93, *p* = 0.002, N = 8).

## Discussion

This study has successfully demonstrated the application of 7T H-1 MRSI to healthy controls and to patients with MDD before and after treatment with MBCT. As reported by Eisendrath et al. [12] for the larger study that our population was drawn from, MBCT greatly reduced the severity of depression. Changes in metabolite levels after completing MBCT treatment, and the correlation between metabolite levels and depression severity are of interest for future use in evaluating individuals who may benefit from this or other, similar therapies. The main finding in this respect is that differences in metabolite ratios observed in patients at baseline were normalized after treatment and became similar to the levels in healthy controls. To our knowledge, this is the first study showing differences in metabolites in patients with MDD who were treated with MBCT alone.

The improved sensitivity and spectral resolution of the 7T scanner is important for optimizing data quality using in vivo MRSI [8, 22]. The method used in this study to estimate metabolite levels was demonstrated previously and has been applied in other patient populations [15]. To avoid the time penalty of acquiring an additional 3D MRSI acquisition with unsuppressed water for scaling metabolite signals, we chose to reference levels of the metabolites of interest to the levels of tCr in the same voxel. Prior studies have shown that these acquisition and postprocessing methods are able to provide uniform and reliable quantification in the entire spectral array for metabolite ratios, including tCho/tCr, NAA/tCr, Glu/tCr, Gln/tCr, GSH/tCr, mI/tCr, Gly/tCr, and GABA/tCr, within a clinically reasonable total acquisition time of ~10 min [15].

Previous studies evaluating metabolites in MDD used a single-voxel MRS acquisition with a large voxel size and produced variable results [10]. This may be due to differences in the patient population or in tissue components studied and the degree of partial volume that was present. Multi-voxel 3D MRSI has the advantage of providing an assessment of spatial distribution of metabolites. In this study, 3D MRSI was prescribed within a 4-cm excitation slab that was parallel to the AC-PC line and had full coverage of the thalamus. Five types of ROIs were defined within frontal and limbic lobes, insular and subcortical regions. The apriori selection of ROIs was limited by the position of the excitation slab such that the cortex was partially suppressed by the eight automatically prescribed outer-volume lipid suppression and the spatial resolution (1 cm^3^) used for data acquisition (see Fig. 1). In an attempt to provide robust measures, we used median metabolite levels from voxels that overlapped by at least 40 % with the anatomic ROIs being considered.

The tCho peak resonates at 3.22 ppm at in vivo spectrum and includes signals from Cho, phosphocholine, glycerophosphocholine, and acetylcholine. Previous studies showed that the level of tCho/tCr was increased in the basal ganglion in patients with MDD [23, 24] and that it decreased with antidepressant treatment (nefazodone) [23]. This is consistent with our findings of significantly increased tCho/tCr in the right caudate in MDD, which decreased to levels in controls after completing MBCT treatment. Although an elevation of tCho/tCr in the basal ganglia has been reported after 8 weeks of fluoxetine treatment in patients who responded to the therapy [25], no controls were included in that study. Elevated tCho is thought to be associated with increased cell density and/or membrane turnover. The alteration in tCho observed in our study suggests abnormalities in myelination [26] and/or glial function [27], which are associated with MDD, and/or altered cellular signal-transduction pathways.

NAA is found only in the brain and spinal cord and is synthesized and stored in neurons but hydrolyzed in oligodendrocytes. Since NAA is a neuronal marker, any pathology with a loss of neurons or function results in reduced NAA levels. Vythilingam et al. [24] reported significantly decreased NAA/tCr in the caudate in patients with MDD. NAA has also been studied in the ACC and the frontal lobe in patients with MDD [28–30], and prior observed that lower NAA in the ACC and decreased NAA/tCr in the MFG at baseline were normalized after antidepressant treatment [30, 31]. This is similar to our results for patients who completed MBCT treatment. Normalization of NAA/tCr within an 8-week treatment period indicates that MDD is more likely to be associated with neuronal dysfunction rather than neuronal loss [32, 33]. We found that significant correlations with percent changes of HAMD-17 scores between baseline and after MBCT treatment were found only for NAA/tCr levels in the left ACC, suggesting that NAA/tCr is a good marker for evaluating response to therapy.

The mI is predominately located within astrocytes and is a precursor for the phosphatidylinositol second-messenger system, which is also presumed to act as an osmoregulator. Significantly decreased mI/tCr levels were previously reported in the frontal lobe in patients with MDD [29, 34], but there have been limited studies of these chemicals in the basal ganglia and insular cortex. The insula plays a role in consciousness and subjective feelings [35], and studies in MDD using functional MRI (fMRI) have detected abnormal interoceptive activity within this region [36]. In our study, decreased mI/tCr was found in the insula in MDD relative to controls, which may indicate glial dysfunction and/or a reduced number of glial cells in patients with MDD [32, 33]. HAMD-17 scores at baseline were significantly correlated with mI/tCr in the insula and putamen, suggesting that mI/tCr may be associated with the severity of depression.

Glu is a main excitatory neurotransmitter in the brain and is converted to Gln by the reabsorption by neurons or reuptake by astrocytes in order to avoid excitotoxicity. It is difficult to separate Glu from Gln in the short TE spectra at 3T, and Glx is typically designated as the sum of Glu and Gln peaks, with Glu as the majority component. A previous study found that Glx was lower in the dorsolateral prefrontal cortex (DLPFC) of MDD patients compared to healthy controls [37] and that Glu was increased in responders after treatment with 10 days of high-frequency transcranial magnetic stimulation (TMS) [38]. Although Glu/tCr in the left caudate at baseline was not significantly different than that in controls in our study (*p* = 0.051), it returned to normal levels after the 8-week treatment with MBCT. Gln is also a precursor for GSH, an antioxidant that prevents damage from reactive oxygen species. The role of GSH in MDD has not been widely studied. A previous study showed no significant correlation between GSH and depression or mania for patients with bipolar disorder [39]. Our findings of decreased GSH/tCr in insula and putamen indicate that GSH may play an important role in MDD.

GABA is a main inhibitory neurotransmitter within the brain. Previous studies reported lower GABA in the occipital cortex in MDD patients [40] and an increase in GABA with antidepressant treatment [41]. Reduction in GABA is possibly due to impaired GABAergic function and/or decreased GABA synthesis [42]. The occipital cortex was not analyzed in our study because it was not covered by the excitation slab. Although decreased GABA was previously reported in ACC [43], there were no significant differences in GABA/tCr in ACC in our study.

This study supports the effectiveness of the MBCT treatment as a monotherapy for acute MDD and the effectiveness of H-1 MRSI to detect metabolite changes associated with successful MBCT treatment for MDD. Although the number of patients was relatively small, a number of key results that are consistent with our understanding of brain chemistry were obtained. Another limitation is that the significant changes in metabolite ratios could have been caused by alteration in tCr level rather than in other metabolites. The tCr, which has peaks at 3.0 and 3.9 ppm, includes Cr and phosphocreatine, which are involved in adenosine triphosphate (ATP) metabolism. Although some previous studies found no significant differences in tCr in patients with MDD [28], others reported a significant increase in tCr compared to controls [34]. Implementing and adding a fast MRSI acquisition for unsuppressed water may be useful to better understand the roles of tCr in MDD, as well as the other metabolites that were observed different in this study. However, our results suggest that using 3D MRSI at 7T in conjunction with metabolite ratios may be a time-efficient strategy for differentiating patients from healthy controls and following changes due to treatment.

## Conclusion

In conclusion, results of our study demonstrate differences in tCho/tCr, NAA/tCr, Glu/tCr, mI/tCr, GABA/tCr, and GSH/tCr in patients with MDD compared to healthy controls and demonstrated changes in these ratios associated with MBCT treatment and reduction in depression severity. Regional differences in metabolite ratios also existed in patients with MDD. These results reflect the complexity of biochemical changes associated with disease processes and treatment effects. Future studies in a larger randomized controlled study population will be helpful for interpreting changes in metabolite ratios and understanding how therapeutic interventions impact MDD. Such information could also be valuable for determining at an early stage whether patients are responding to therapy or whether a new treatment strategy should be considered.
